# The prevalence of gestational diabetes, associated factors and feto-maternal outcome among antenatal women attending health clinics in Terengganu

**DOI:** 10.51866/oal302

**Published:** 2022-07-12

**Authors:** Rozimah Abd Latif, Nurul Azreen Yusof, Ranimah Yahya, Zahrni Muda, Tengku Bahiah Tengku Lih, Kamilah Mohamed, Darisah Lah, Rohaiza Abd Kadir, Maira Hassan, Wan Ruzilasalwa Wan Sulaiman, Siti Aminah Akbar Merican, Mohd Sharil Iman Mohd Hanafi

**Affiliations:** 1 MD (UKM), MMed (Family Medicine) USM, Faculty of Medicine, Universiti Sultan, Zainal Abidin, Kuala Terengganu, Terengganu, Malaysia. Email: azreenyusof@unisza.edu.my; 2 MMBS (Adelaide), MMed (Family Medicine) USM, Klinik Kesihatan Merchang, Marang, Terengganu, Malaysia.; 3 MD (UKM), MMed (Family Medicine) USM, Klinik Kesihatan Rahmat, Chalok, Setiu, Terengganu, Malaysia.; 4 MD (USM), MMed (Family Medicine) USM, Klinik Kesihatan Al-Muktafi Billah, Shah, Jengai, Dungun, Terengganu, Malaysia.; 5 MD (UKM), MMed (Family Medicine) UKM, Klinik Kesihatan Padang Luas, Jerteh, Besut, Terengganu, Malaysia.; 6 MD (UKM), MMed (Family Medicine) UKM, Klinik Kesihatan Batu Rakit, Kuala, Nerus, Terengganu, Malaysia.; 7 MD (UKM), MMed (Family Medicine), UKM, Klinik Kesihatan Bukit Tunggal, Kuala, Nerus, Terengganu, Malaysia.; 8 MD (UKM), MMed (Family Medicine) UKM, Klinik Kesihatan Seberang Takir, Kuala Nerus, Terengganu, Malaysia.; 9 MBBS (Banglaore), MMed (Family Medicine) USM, Klinik Kesihatan Manir, Kuala, Terengganu, Terengganu, Malaysia.; 10 MD (UKM), MMed (Family Medicine) UKM, Klinik Kesihatan Kuala, Dungun, Kg Alor Tembese, Kuala Dungun, Dungun, Terengganu, Malaysia.; 11 MD (USM), MMed (Family Medicine) UKM, Klinik Kesihatan Kuala, Berang, Kuala Berang, Terengganu, Malaysia.; 12 MBBS (UniSZA), Klinik Kesihatan Merchang, Marang, Terengganu, Malaysia.

**Keywords:** Gestational diabetes mellitus, Risk factors, Foeto-maternal outcomes

## Abstract

**Introduction:**

Gestational diabetes mellitus (GDM) is a known risk factor for diabetes mellitus (DM). The rising prevalence of GDM in the Asian population (11.7%) may explain the increasing incidence of DM in women. This study examined the prevalence of GDM, its associated factors and the foeto-maternal outcomes of women with GDM in Terengganu.

**Method:**

A cross-sectional study was conducted between April and September 2019 using secondary data from antenatal records in 40 health clinics in Terengganu for 2018. All pregnant women aged 25 years and above with or without risk factors for GDM were included in the study. Those with pre-existing type 1 or 2 DM were excluded. A total of 270 respondents were included. The prevalence of GDM and its associated factors were determined using descriptive statistics followed by multiple logistic regression.

**Results:**

The prevalence of GDM in Terengganu was 27.3% (n=72). Logistic regression analysis found that BMI at booking (adjusted OR=4.51, 95% CI 2.13–9.55, p<0.001), history of GDM (adjusted OR=5.31, 95% CI 2.17–12.99, p<0.001) and family history of DM (adjusted OR=4.24, 95% CI 2.23–8.05, p<0.001) were the significant associated risk factors. Of women with GDM, 17.7% (n=11) had postpartum pre-diabetes based on modified oral glucose tolerance at 6 weeks postpartum. Univariate analysis using chi-square tests showed a significant association of neonatal jaundice and hypoglycaemia with GDM.

**Conclusion:**

Because the prevalence of GDM in Terengganu is high, surveillance of GDM in highrisk pregnancies and effective glycaemic management should be emphasised to prevent adverse foeto-maternal outcomes.

## Introduction

Gestational diabetes mellitus (GDM) is a state of glucose intolerance of varying degrees with onset or first recognition during pregnancy.^1^ It has been associated with short-term and long-term adverse outcomes for both mothers and newborns.^2^ The National Health Morbidity Survey (NHMS) 2015 showed a prevalence of diabetes mellitus (DM) among adult women in Malaysia of 18.3%.^3^ One risk factor for developing DM is gestational diabetes mellitus (GDM). Asia has an increasing prevalence of GDM, which varies widely depending on the population studied and the diagnostic test employed.^4^ According to a meta-analysis, the pooled prevalence of GDM was 11.5% in Asia and 10.1% in Southeast Asia.^5,6^ However, a local study in Selangor in 2003 reported a higher prevalence of GDM (18.3%),^7^ and this was even higher in another local study 11 years later (27.9%).^8^ Other studies have found the prevalence ranges from 2.4% to 21.0%.^9,10^

Based on current guidelines, the diagnosis of GDM is based on a fasting plasma glucose (FPG) value of more or equal to 5.1 mmol/L and/or a 2-hour post-prandial (2HPP) value of more or equal to 7.8 mmol/L.^11^ In Terengganu, this guideline was applied to the whole state in 2019. Since this study was conducted in 2018, the diagnosis of GDM was based on the previous guideline, which was an FPG of 5.6 mmol/L and a 2HPP of 7.8 mmol/L.^12^ The most commonly reported risk factors for GDM were older age, prepregnancy obesity, high parity, family history of diabetes (especially in first-degree relatives), previous history of GDM, and previous obstetric outcomes such as a macrosomic infant, congenital malformation and recurrent abortions. Maternal overweight or obesity and family history of GDM were recognised as established risk factors for GDM.^7,13^ Women with GDM were also at increased risk of caesarean section, gestational hypertension, preeclampsia and type 2 DM.^14,15^ The newborns of mothers with GDM have been associated with macrosomia or larger than normal gestational age, neonatal hypoglycaemia and type 2 DM later in life.^5,16,17^

Given the rapid socioeconomic and nutrition transition and the increasing prevalence of GDM in Asia,^18^ it is important to recognise the risk factors among the local population so that screening systems and prepregnancy management can be strengthened. Additionally, the prevalence of GDM and foeto-maternal outcomes in Terengganu have never been reported. Hence, the results of this study will be the reference and basis for future studies of mothers with GDM, especially in exploring modifiable risk factors and the association of GDM with foeto-maternal outcomes to reduce its morbidity and mortality.

## Methods

This cross-sectional study was conducted between April and September 2019. It involved antenatal women who attended health clinics and delivered in Terengganu state in 2018. Based on the current guideline, women with risk factors for GDM were screened using a modified oral glucose tolerance test (MOGTT) at booking or as early as possible. If the initial result was normal, a second MOGTT was done between 24 and 28 weeks’ gestation. The inclusion criteria were antenatal women aged 25 years and above with or without risk factors for GDM as recommended by the local guideline. Those with pre-existing type 1 or 2 DM were excluded. The sample size was calculated using OpenEpi software. The sample size was calculated based on the parameters of population size: 1,000,000; anticipated frequency: 18.3%^7^; absolute precision: 0.05; design effect: 1. The calculated sample size was 226. After considering 20% drop-out, 283 was taken as the sample size for this study.

Of the total of 40 health clinics located in eight districts in Terengganu, all were involved in this study. The total sample size was then allocated proportionately to eight districts in Terengganu based on the percentage of total deliveries in each district. For each clinic, samples were selected using stratified random sampling generated by an online random number generator. Antenatal record books of selected cases were retrieved from respective health clinics. The retrieved information was for the dependent variable (GDM) and the independent variables, including sociodemographic data (age, education level, occupation, family history of DM), obstetric factors (gravida, trimester at booking, BMI at booking, weight gain of more than 2 kg/ week, recurrent urinary tract infection), past obstetric history (previous macrosomia, history of GDM, history of abortions, intrauterine death), investigations (HbA1c result, postnatal MOGTT results) and foeto-maternal outcomes (delivery method, neonatal jaundice, hypoglycaemia, foetal macrosomia, respiratory distress syndrome [RDS], preterm delivery). A proforma was applied to record all of this data.

The data were analysed using IBM SPSS ver. 20.0 (IBM Corp., Armonk, NY, USA). Descriptive analysis was performed, in which categorical variables were presented as frequencies (percentages). The prevalence of GDM was described as frequencies (percentages). Simple and multiple logistic regression analyses were used to determine factors associated with GDM among pregnant women. Simple logistic regression was used for screening in the selection of variables, whereby all variables with a p-value of less than 0.25 and clinically significant variables were then included in multiple logistic regression. Forward and backward logistic regression methods were used, where the selection of variables was based on Wald’s test. This process of deleting, refitting and verifying continued until all the important variables appeared included in the model and those excluded were clinically or statistically unimportant. At this step, the preliminary main effect model was obtained. All possible two-way interactions were checked. The independent variables were fitted into multiple logistic regression, and variance inflation factors were obtained to check for multicollinearity. Multicollinearity was present if the VIF values were greater than 5. The findings were presented with the crude and adjusted odds ratio (OR), 95% confidence interval (CI), and p-value. The level of significance was set at 0.05 in a two-tailed fashion. Univariate analysis using chi-square tests (Fisher’s exact test when applicable) was used to find the associations between GDM and the foeto-maternal outcomes.

This study was approved by National Medical Research Register (NMRR) committee (NMRR-19-1527-46454[IIR]).

## Results

### i) Sociodemographic and clinical characteristics of GDM among antenatal women

Of the reviewed antenatal records, 264 (93.2%) were completed in proforma and hence included in the analysis. The characteristics of pregnant women in this study are presented in **Table 1**. The majority of respondents were Malay (n=261, 98.9%), were aged under 35 years (n=193, 73.0%) and had a secondary education (n=164, 62.0%). Most were multiparous (n=158, 59.8%) and had early bookings in the first trimester (n=213, 80.7%).

### ii) Prevalence of GDM

The prevalence of GDM in this study was 27.3% (n=72). The prevalence of GDM was higher in those with a family history of DM (n=36, 48.6%) and a history of GDM (n=19, 65.5%). The other associated factors are described in **Table 1**. Of the women with GDM, 95.8% (n=69) had good baseline glycaemic control, with an HbA1c less than or equal to 6.5%. All were advised about diet control (n=72, 100%), almost all received dietary counselling by the dietitian (n=69, 95.8%) and only 22.2% were started on insulin therapy.

**Table 1:** Sociodemographic and clinical characteristics of GDM patients (n=264).

Variables	n (%)	No GDM (n=192)	GDM (n=72)
**Sociodemographic characteristics:**			
*Age group*			
Less than 35 years old	193 (73.1)	149 (77.2)	44 (22.8)
35 years old and above	71 (26.9)	43 (60.6)	28 (39.4)
*Ethnic group*			
Malay	261 (98.9)	191 (73.2)	70 (26.8)
Chinese	3 (1.1)	1(33.3)	2 (66.7)
*Education level*			
No formal/primary education	8 (3.0)	6 (75.0)	2 (25.0)
Secondary education	164 (62.1)	123 (75.0)	41 (25.0)
Tertiary education	92 (34.8)	63 (68.5)	29 (31.5)
*Occupation*			
Working	117 (44.3)	87 (74.4)	30 (25.6)
Home duties	147 (55.7)	105 (71.4)	42 (28.5)
*Family history of DM*			
No	190 (72.0)	154 (81.1)	36 (18.9)
Yes	74 (28.0)	38 (51.4)	36 (48.6)
**Obstetric characteristics:**			
*Gravida*			
Primigravida	58 (22.0)	47 (81.0)	11 (19.0)
Multipara	158 (59.8)	114 (72.2)	44 (27.8)
Grand multipara	48 (18.2)	31 (64.6)	17 (35.4)
*Trimester at booking*			
First trimester	213 (80.7)	156 (73.2)	57 (26.8)
Second/third trimester	51 (19.3)	36 (70.6)	15 (29.4)
*BMI at booking*			
Normal	99 (37.5)	88 (88.9)	11 (11.1)
Overweight/obese	165 (62.5)	104 (63.0)	61 (37.0)
*Weight gain >2 kg/week*			
No	237 (89.8)	174 (73.4)	63 (26.6)
Yes	27 (10.2)	18 (66.7)	9 (33.3)
*Urinary tract infection/candidiasis*			
No	252 (95.8)	185 (73.4)	67 (26.6)
Yes	11 (4.2)	7 (63.6)	4 (36.4)
**Past obstetric history:**			
*Previous macrosomic baby*			
No	253 (95.8)	185 (73.1)	68 (26.9)
Yes	11 (4.2)	7 (63.6)	4 (36.4)
*History of GDM*			
No	235 (89.0)	182 (77.4)	53 (22.6)
Yes	29 (11.0)	10 (34.5)	19 (65.5)
*History of abortion*			
No	256 (97.0)	188 (73.4)	68 (26.6)
Yes	8 (3.0)	4 (50.0)	4 (50.0)
*History of intrauterine death*			
No	262 (99.2)	191 (72.9)	71 (27.1)
Yes	2 (0.8)	1 (50.0)	1 (50.0)
**Investigations:**			
*HbAlc level*			
6.5% and below			69 (95.8)
Greater than 6.5%			3 (4.2)
*Postnatal MOGTT*			
Normal			51 (82.3)
IFG/IGT			11 (17.7)
Diabetes			0
**GDM management:**			
*Diet control*			
No			72 (100)
Yes			0
*Insulin initiation*			
No			16 (22.2)
Yes			56 (77.8)
*Dietitian counselling*			
No			69 (95.8)
Yes			3 (4.2)

### iii) Factors associated with GDM

Simple logistic regression showed a significant association of GDM with age, gravida, BMI at booking, family history of DM, history of GDM and history of abortion (**Table 2**).

**Table 2:** Simple logistic regression of factors associated with GDM (n=264).

Significant variables	B^a^	Crude OR^b^	95% CI^c^	Wald stat^d^ (df)	p-value
*Age*					
35 years or more	0.79	2.21	1.23-3.95	7.07(1)	0.008
Less than 35 years		1.00			
*Gravida*				3.58 (2)	0.167
Grand multipara	0.85	2.34	0.97 5.67	3.57(1)	0.059
Multipara	0.50	1.65	0.79-3.47	1.74(1)	0.187
Prirnigravida		1.00			
*BMI at booking*			
Over weight/obese	1.55	4.70	2.33-9.47	18.63(1)	<0.001
Normal		1.00			
*Weight gain >2 kg/week*					
Yes	0.32	1.38	0.59-3.23	0.55(1)	0.457
No		1.00			
*Family history of GDM*					
Yes	1.40	4.05	2.26 7.26	22.16(1)	<0.001
No		1.00			
*History of GDM*					
Yes	1.88	6.53	2.81-14.88	19.87 (1)	<0.001
No		1.00			
*History of abortion*					
Yes	1.02	2.77	0.67-11.36	1.99 (1)	0.158
No		1.00			
*History of intrauterine death*					
Yes	0.99	2.69	0.17-43.59	0.49 (1)	0.486
No		1.00			
*Recurrent UTI*					
Yes	0.44	1.56	0.44-5.48	0.47 (1)	0.492
No		1.00			

a Regression coefficient

b Adjusted odds ratio

c 95% confidence interval

d Wald statistic (degree of freedom)

Multiple logistic regression showed that BMI at booking, family history of DM and history of GDM were significant associated factors of GDM when other confounders were controlled (**Table 3**).

**Table 3:** The association between GDM and the risk factors of GDM by multiple logistic regression (n=264).

Significant variables	B^a^	Adjusted OR^b^	95% CI^c^	Wald staf^d^ (df)	p-value
*BMI at boohing*					
Overweight/obese	1.51	4.51	2.13-9.55	15.44(1)	<0.001
Normal		1.00			
*Family history of DM*					
Yes	1.44	4.24	2.23 8.05	19.42(1)	<0.001
No		1.00			
*History of GDM*					
Yes	1.67	5.31	2.17-12.99	13.34(1)	<0.001
No		1.00			

a Regression coefficient

b Adjusted odds ratio

c 95% confidence interval

d Wald statistic (degree of freedom)

Constant value : -2.762. Forward and backward method applied. No multicollinearity present. No significant interaction found. Hosmer—Lemeshow test, p-value 0.550. Percentage of classification table correctly classified was 72.7%. Percentage of area under receiver operating characteristics (ROC) curve was 0.759.

In the final model, women who were overweight or obese at booking had 4.51 times greater odds of having GDM than those with a normal BMI (adjusted OR=4.51, 95% CI 2.13-9.55, p<0.001). Similarly, those with a family history of DM had 4.24 times greater odds of having GDM compared to those without a family history of DM (adjusted OR=4.24, 95% CI 2.23-8.05, p<0.001). Antenatal women with a history of GDM had 5.31 times greater odds of having GDM in the current pregnancy than those without a history of GDM (adjusted OR=5.31, 95% CI 2.17-12.99, p<0.001).

### iv) Foeto-maternal outcomes

The associations between GDM and foeto-maternal outcomes are summarised in **Table 4**. For maternal outcomes, no significant association existed between GDM and the method of delivery. For foetal outcomes, a significant association existed between GDM and neonatal jaundice as well as neonatal hypoglycaemia. No significant association with macrosomia, RDS or preterm delivery was observed.

**Table 4:** The association between GDM and adverse foeto-maternal outcomes (n=72).

Foeto-maternal outcome		GDM status		X^2^ statistic (df)	p-value Fisher’s exact test^*^
		No GDM, n(%) (n=192)	GDM, n(%) (n=72)		
**Maternal outcome:**					
*Delivery method*					
SVD	No	32 (65.3)	17 (34.7)		
	Yes	160 (74.4)	55 (25.6)	1.67 (1)	0.196
LSCS	No	161 (74.5)	55 (25.5)		
	Yes	31 (64.6)	17 (35.4)	1.96 (1)	0.161
Instrumental delivery	No	191 (72.6)	72 (27.4)		
	Yes	1 (100.0)	0		0.727*
**Foetal outcome:**					
Neonatal jaundice	No	96 (78.7)	26 (21.3)	4.06 (1)	**0.044**
	Yes	96 (67.6)	46 (32.4)		
Hypoglycaemia	No	191 (73.7)	68 (26.3)		0.021*
	Yes	1 (20.0)	4(80.0)		
Macrosomia	No	189 (72.7)	71 (27.3)		0.700*
	Yes	3 (75.0)	1 (25.0)		
RDS	No	187 (72.8)	70 (27.2)		0.613*
	Yes	5 (71.4)	2 (28.6)		
Preterm delivery	No	189 (73.0)	70 (27.0)		0.616*
	Yes	3 (60.0)	2 (40.0)		

SVD — spontaneous vaginal delivery LSCS — lower segment caesarean section RDS — respiratory distress syndrome

## Discussion

### Prevalence of GDM

The prevalence of GDM in clinics in Terengganu was 27.3%, which is comparable with a study in public health clinics in Selangor in 2017 (27.9%).^8^ However, the prevalence of GDM was lower in other studies locally^7^ and internationally, ranging from 6.6% to 18.3%.^5,13,19^ A 2018 metaanalysis by Lee et al.^6^ found that the pooled prevalence of GDM was 11.5% (95% CI 10.9–12.1). This is considered more representative of the burden of GDM across Asian populations. This prevalence of GDM in Asia is higher than in European countries (5.4%) but lower than in African countries (14.0%). This might be due to differences in maternal age, BMI disparities and ethnic backgrounds.^6^

Over the years, additional risk factors included in GDM screening have resulted in higher diagnosis rates. Previously, based on World Health Organization 1998 diagnostic criteria, for those with risk factors, screening was done once at 24 to 28 weeks of gestation. From 2008, GDM screening was recommended to be done twice.^8^ Later, the NICE 2015 guideline introduced universal screening of women aged more than 25 years without other additional risk factors at 24 to 28 weeks’ gestation.^12^ Other possible contributors are modifiable risk factors for GDM, such as high carbohydrate intake, sedentary lifestyle and smoking, which vary between regions and countries. This explains the different rates of glucose intolerance among different populations, which then leads to pancreatic B-cell exhaustion in pregnancy.^20^ However, these factors were not included in this study.

### Associated Factors of GDM

BMI at booking, family history of DM and history of GDM were found to be significant in our study. We found that the odds of GDM increased 4.51 times among overweight or obese women compared to those with a normal BMI (adjusted OR=4.51, 95% CI 2.13-9.55, p<0.001). This finding is consistent with numerous studies that have reported an increased risk of GDM among women who are overweight or obese compared with lean or normal-weight women. Based on a meta-analysis on maternal obesity and the risk of GDM, the unadjusted ORs of GDM were 2.14 times higher among overweight women, 3.56 times higher among obese women and 8.56 higher among severely obese women compared with normal-weight pregnant women.^4^ Priyanka et al. also found a significant association between maternal obesity and GDM.^19^ In obese women, the pathophysiology is primarily characterised by the pregnancy-induced insulin resistance being amplified by the already elevated prepregnant insulin resistance level. The elevated insulin resistance level is a known factor in the metabolic syndrome. These defects disrupt insulin action in maintaining glucose levels, resulting in maternal hyperglycaemia.^21^

Another significant finding was that those with a family history of DM had 4.24 times greater odds of having GDM compared to those without a family history of DM (adjusted OR=4.24, 95% CI 2.23-8.05, p<0.001). Similarly, a systematic review^6^ found that those with a family history of diabetes had 2.77 times higher odds of having GDM. Other studies^8,13,17,19^ obtained similar findings. Researchers have identified various genetic variants appearing to impact B-cell function, explaining the theory of genetic predisposition to diabetes.^13^ A metaanalysis also found that the odds of GDM were increased by 8.42 times in those with a previous history of GDM compared to those with no history of GDM. A similar association was found by other studies.^8,13,19^ These are consistent with our finding that antenatal women with a history of GDM had 5.31 times greater odds of having GDM in the current pregnancy than those without a history of GDM (adjusted OR=5.31, 95% CI 2.17-12.99, p<0.001). Based on our extensive search of available studies in the medical literature, none had contradicting results. Those with a history of GDM were found to have abnormal carbohydrate metabolism and pancreatic P-cell function and insulin insensitivity.^5,8^

Nevertheless, the other risk factors for GDM (poor obstetric history, history of macrosomia, history of abortion, polyhydramnios, urinary tract infection and weight gain of more than 2 kg/week) were statistically not significant in our study. A possible reason is that the majority of women with GDM in our study had good glycemic control, as evidenced by 95.8% (n=69) having an HbA1c less than or equal to 6.0%, resulting in a lesser degree of GDM complications.

### Foeto-Maternal Outcomes

GDM has a well-known association with an increased rate of caesarean section. The common direct causes are acute foetal distress, failed induction of labour and macrosomia.^22^ The proportion of caesarean section among women with GDM in our study was 35.4% (n=17), which is comparable to the national prevalence of 34.5% to 35.5% (from 2013-2015).^22^ However, the proportion of caesarean section among women with GDM was higher in a prospective study in India (79.0%), with the most common indication being arrest of labour.^19^ Other studies have shown varying rates of caesarean section for women with GDM, ranging from 18.0% to 28.5%.^8,20^ This difference is probably due to the majority having good diabetic care and controlled diabetes and thus less macrosomia and labour arrest that contribute to caesarean section. Other possibilities are the different clinical settings, adequacy of intrapartum foetal monitoring and surveillance. The complications that might happen due to uncontrolled GDM can be minimised by proper glucose control throughout pregnancy, which reduces the caesarean section rate. These factors might be why our study found no significant association between GDM and the delivery method. In this study, 95.8% (n=69) of mothers with GDM underwent nutritional therapy by a dietitian, and 22.2% (n=16) were started on insulin to ensure proper glucose optimisation. The other study used a similar management approach, whereby 42.4% of those with uncontrolled GDM were started on insulin to improve their glycaemic control.^13^

A systematic review of 20 studies found at least a 7-fold increase in the risk of developing type 2 DM, when comparing women with a pregnancy complicated by GDM to women with a normoglycemic pregnancy.^21^ Therefore, post-partum diabetic screening is recommended for all women with GDM. In clinical practice, postnatal glycaemic status needs to be confirmed with a repeat MOGTT at 6 weeks postpartum.^12^ In our study, of the 86% (n=62) of women with GDM who underwent MOGTT postnatally, 17.7% were diagnosed with pre-diabetes. The prevalence of postpartum pre-diabetes was higher than in a 2014 local study in Selangor (12.1%).^14^ This difference in the prevalence of abnormal postnatal MOGTT may be due to the differences in ethnicity involved, as well as the rate of weight gain during pregnancy among women with GDM, which was greater in our study compared to the study in Selangor (33.3% vs 20.7%).^8^

Further, in our study, 48.6% of women with GDM had a family history of DM, and 65.5% had a history of GDM (compared to the study in Selangor: 38.3% had a family history of DM, and only 16.2% had a history of GDM), which contributed significantly to the risk of developing pre-diabetes as well as diabetes postpartum. The prevalence of DM was observed in the systematic review to range from 2.6% to 70% at 6 weeks or even 28 years postpartum. Most studies have reported the cumulative incidence of type 2 DM. Knowing diabetic status in the postpartum period allows early treatment and close follow-up, given the future diabetic risk. Furthermore, comprehensive prepregnancy counselling can reduce maternal and foetal morbidity and mortality. Although none of our women were diabetic during postpartum, they must be aware of this risk, and lifestyle modification right after delivery is recommended to minimise the future risk of diabetes.

For foetal outcomes, our study found a significant association between GDM and hypoglycaemia. A retrospective study in Sydney in 2019 showed similar associations; the risk of developing hypoglycaemia increased 1.8-fold in pregnancies complicated with GDM.^23^ However, a study in Selangor in 2017 did not find such associations.^8^ Neonatal hypoglycaemia is associated with elevated levels of maternal glucose, resulting in foetal hyperinsulinemia, which leads to hypoglycaemia in infants.^24^ Therefore, preconception care and antenatal optimisation of glycaemic control could yield reductions in adverse neonatal outcomes.^23^ Our findings suggest that GDM was associated with neonatal hyperbilirubinemia. This is supported by a 2019 study that found that the risk of neonatal hyperbilirubinemia increased 1.5-fold in pregnancies complicated with GDM.^22^ However, Logakodie et al. did not find such an association.^8^ National data in 2015 reported that mothers with GDM had higher rates of macrosomic infants and preterm birth, similar to another study.^19,22^ However, we did not find these factors to be significantly associated with GDM.

### Study Strengths and Limitations

This study was conducted at all clinics in rural and urban areas throughout Terengganu. Hence, it could represent the pregnant population in this state. However, the prevalence of GDM in this study might be lower than the actual prevalence because we used the former diagnostic criteria. The GDM criteria used were an FPG value of more than or equal to 5.6 mmol/L or a 2HPP of more than or equal to 7.8 mmol/L, as in 2018. Hence, the prevalence might be higher if we used the current diagnostic criteria stated in our national guidelines. Another limitation is that this study did not explore the modifiable risk factors for GDM, such as lifestyle practices, or other foeto-maternal outcomes commonly associated with GDM, such as gestational hypertension, preterm prelabour membrane rupture, shoulder dystocia and stillbirth because these data were not available in the reviewed records. Future study is needed to examine these associations so that modifiable risk factors can be identified and tackled before pregnancy to minimise the risk of GDM. The association between maternal glycaemic control and foeto-maternal outcomes is another area to be explored.

## Conclusion

The high prevalence of GDM among antenatal women in Terengganu has raised the concern of future increased incidence of DM in not only the women but also their offspring. However, with optimised maternal glycaemic control, adverse foeto-maternal outcomes have been reduced over time. Surveillance of GDM in high-risk antenatal mothers and effective glycaemic control should be emphasised to prevent adverse foeto-maternal outcomes.

**How does this paper make a difference in general practice?**

- This study provides baseline data on GDM prevalence and its risk factors in Terengganu. Future study is needed to prospectively see the trend of future development of DM and maternal modifiable risk factors.
- The two significant risk factors found in the study (family history of DM and history of GDM) warrant education and early diet intervention for women with such risks.
- This study found a lower rate of foeto-maternal complications compared to other studies, which indicates good surveillance and proper management of GDM were applied in clinical practice. These must be emphasised to further reduce foeto-maternal morbidity and mortality.
